# Effects of Atrial Natriuretic Peptide on Bicarbonate Transport in Long- and Short-Looped Medullary Thick Ascending Limbs of Rats

**DOI:** 10.1371/journal.pone.0083146

**Published:** 2013-12-23

**Authors:** Hiroshi Nonoguchi, Yuichiro Izumi, Yushi Nakayama, Takanobu Matsuzaki, Yukiko Yasuoka, Takeaki Inoue, Hideki Inoue, Tomohiko Mouri, Katsumasa Kawahara, Hideyuki Saito, Kimio Tomita

**Affiliations:** 1 Department of Internal Medicine and Education & Research Center, Kitasato University Medical Center, Kitamoto, Saitama, Japan; 2 Systems Biology Center, National Heart, Lung and Blood Institute, National Institutes of Health, Bethesda, Maryland, United States of America; 3 Department of Nephrology, Kumamoto University Graduate School of Medical Sciences, Kumamoto, Kumamoto, Japan; 4 Department of Pharmacy, Kumamoto University Hospital, Kumamoto, Kumamoto, Japan; 5 Department of Physiology, Kitasato University School of Medicine, Sagamihara, Kanagawa, Japan; Aarhus University, Denmark

## Abstract

Atrial natriuretic peptide (ANP) is known to influence NaCl transport in the medullary thick ascending limbs (MAL), where the largest NaCl reabsorption occurs among distal nephron segments in response to arginine vasopressin (AVP). In the present study, we investigated the effect of ANP on bicarbonate (HCO_3_^−^) transport in the MAL using an isolated tubule perfusion technique. The HCO_3_^−^ concentration was measured using free-flow ultramicro-fluorometer. We first observed basal HCO_3_^−^ reabsorption in both long- and short-looped MALs (lMALs, and sMALs, respectively). AVP inhibited HCO_3_^−^ reabsorption in both lMALs and sMALs, whereas ANP did not change HCO_3_^−^ transport. However, in the presence of AVP, ANP restored the HCO_3_^−^ reabsorption inhibited by AVP both in lMAL and sMAL. The effects of ANP on HCO_3_^−^ transport was mimicked by cyclic GMP. The mRNA expression level of the vasopressin V2 receptor in lMALs was significantly higher than in sMALs, whereas expression of the V1a receptor was unchanged. In summary, AVP inhibits HCO_3_^−^ transport, and ANP counteracts the action of AVP on HCO_3_^−^ transport both in lMALs and sMALs.

## Introduction

Arginine vasopressin (AVP) plays a central role in urine concentration and dilution by the kidney [1]–[3]. AVP is known to stimulate NaCl reabsorption in the medullary thick ascending limbs (MAL) where AVP-stimulated Cl reabsorption is highest among the distal nephron segments [4]–[8]. Because water is not absorbed in MALs, they are considered a diluting segment [5], [6], [9]. There are two types of nephrons: long- and short-looped nephrons [9], [10], which are classified according to their long- and short-looped MALs (lMALs and sMALs, respectively). The functional differences between lMALs and sMALs are not well known [10], [11]. The proportion of lMALs and sMALs differs among animals [10]. Humans have a larger number of sMALs than lMALs. In contrast, rats and mice have a larger number of lMALs than sMALs. The pocket mouse has a 10-fold higher single-nephron glomerular filtration rate via long-looped nephrons compared with short-looped nephrons. We have previously shown that AVP-stimulated NaCl reabsorption occurs only in lMALs not in sMALs [11]. It appears that lMALs have a more important role in urine concentration than do sMALs [10].

The kidney plays a major role in not only NaCl and water reabsorption but also in acid excretion [12]. Acid excretion by the kidney consists of bicarbonate (HCO_3_^−^) reabsorption along the whole nephron and ammonia and titratable acid excretion in the distal nephron. The MAL has a large HCO_3_^−^ absorptive ability and AVP inhibits this abikity [13]–[18]. The effect of AVP on Cl transport is different between lMALs and sMALs, suggesting heterogeneity of NKCC2 in lMALs and sMALs [11]. However, it is not known whether the effects of AVP on HCO_3_^−^ transport are different between lMALs and sMALs.

Intravenous administration of atrial natriuretic peptide (ANP) is also known to increase NaCl excretion by stimulating guanylate cyclase dependent-cGMP accumulation across almost all nephron segments [19]–[21]. We have previously shown that ANP counteracts the stimulatory effect of AVP on Cl^−^ reabsorption in lMALs but not in sMALs [11]. However, it is not known whether ANP influences HCO_3_^−^ transport in lMALs and sMALs.

The aim of our study was to investigate the effects of AVP and ANP on HCO_3_^−^ transport in lMALs and sMALs. We also examined the mRNA expressions of vasopressin V1a and V2 receptors in lMALs and sMALs using real-time PCR.

## Methods

### Ethics Statement

The protocols for all of the animal experiments were reviewed and approved by the Committee for Animal Experiments at Kitasato University Medical Center (25-2) and Kumamoto University Graduate School of Medical Sciences (18-059, 19-063).

### Microperfusion of lMALs and sMALs

Both lMALs and sMALs were dissected from male pathogen-free Sprague-Dawley rats weighing 60–100 g as previously described [11]. In brief, an lMAL was confirmed by the attachment of a thin ascending limb from the inner medulla. A sMAL was thicker than an lMAL and was confirmed by the attachment of a thin descending limb, which comes from the outer stripe of the outer medulla. A lMAL has a straight end whereas a sMAL has a rounded end. Only one tubule (lMAL or sMAL) was obtained from one rat considering the viability of the tubules. Thus the sample number *n* indicates the number of perfused tubules and rats used. The dissection solution had the following composition (in mM): 130 NaCl, 5 KCl, 1 NaH_2_PO_4_, 1 MgSO_4_, 1 Ca lactate, 2 Na acetate, 5.5 glucose, 5 L-alanine, 2 L-leucine, 10 HEPES; the pH was adjusted to 7.4 by adding NaOH (final composition: Na^+^ 133, K^+^ 5, Cl^−^ 135, Ca^2+^ 1, Mg^2+^ 1, H_2_PO_4_^−^ 1, SO_4_^2−^ 1, lactate^−^ 1, acetate^−^ 2, alanine^−^ 5, leucine^−^ 2, glucose 5.5, HEPEs 10).

To examine the effects of AVP or ANP on thetransepithelial potential difference (PD) and HCO_3_^−^ reabsorption, the tubule was washed for at least 20–30 min after starting microperfusion to remove intrinsic AVP. After two to three control collections were performed, AVP or ANP was added to the bath solution. From two to five experimental collections were started after 15 min, and then the AVP or ANP was removed from the bath. From two to four recovery collections were made after 20 min of washout.

To examine the effects of ANP in the presence of AVP, AVP was added to the bath from the beginning. Control collections were made 30 min after the start of the perfusion. Then, ANP was added to the bath and experimental collections were performed after 15 min. Recovery collections were made 20 min after the washout of ANP.

The perfusion solution and the bath solution were identical and the following composition (in mM): 118 NaCl, 25 NaHCO_3_, 2.5 K_2_HPO_4_, 1 MgSO_4_, 1 Ca lactate, 2 Na acetate, 5.5 glucose, 5 L-alanine, 2 L-leucine (final composition: Na^+^ 145, K^+^ 5, Cl^−^ 118, Ca^2+^ 1, HCO_3_^−^ 25, HPO_4_^2−^ 2.5, SO_4_^2−^ 1, acetate^−^ 2, alanine^−^ 5, leucine^−^ 2, glucose 5.5). The solution was continuously gassed with 5% CO_2_-95% O_2_.

### Measurement of PD and HCO_3_^−^

The PD was continuously measured using calomel electrodes and agarose bridges (0.16 M NaCl). Agarose bridges were connected to the end of the perfusion pipette (perfusate) and the bath. The total CO_2_ concentration is the sum of the bicarbonate, carbonate and dissolved CO_2_ levels. Because the dissolved CO_2_ is very small in amount, the bicarbonate concentration can be considered the total CO_2_ concentration. The HCO_3_^−^ concentrations in perfusate, bath, and collected fluid were measured using free-flow ultramicro-fluorometer as described previously [22]. The reagent for the determination of T_CO2_ (Sigma 130-UV) was protected from T_CO2_ in the air with oil at the injection port and CO_2_ absorber (Ascarite II, Thomas Scientific, NJ) behind the reservoir of the reagent. The otal CO_2_ concentration in the perfusate was repeatedly measured during the experiments to check the stability of the solution. Because there was no fluid transport in the lMAL and sMAL as we reported previously, the net bicarbonate transport (J T_CO2_) was calculated as J T_CO2_ = (C_O_-C_L_)V_L_/L, where C_O_ and C_L_ are the HCO_3_^−^ concentration in the collected fluid and perfusate, respectively, V_L_ is the rate at which fluid is collected at the end of the tubule, and L is the tubule length. The perfusate and bath solutions were continuously babbled with 95% O_2_ - 5% CO_2_. The tubules were perfused by identical perfusate and bath solutions. Because dissolved CO_2_ is very small in amount and the viability of the perfused tubules was checked against the PD, any leak of CO_2_ from the cell membrane can be ruled out. Because we could measure bicarbonate transport and the values were very close to the values reported by Good et al., we considered that our measurements of the bicarbonate would be accurate. To avoid CO_2_ loss into the mineral oil in the collection pipette, the mineral oil was water-saturated, and the water was bubbled with 95% O_2_ - 5% CO_2_ before the experiments.

### Real-time PCR with microdissected nephron segments

Isolation of mRNA from the renal tubules and reverse transcription were performed as described previously [23], [24]. sMAL and lMAL were dissected as described above from control rats after 30 min incubation of kidney slices at 37°C in the 0.1% collagenase solution in the presence of vanadyl ribonucleoside complex (VRC). We performed a TaqMan quantitative real-time RT-PCR using an ABI PRISM 7900 sequence detection system (Applied Biosystems, CA, USA) to determine the mRNA expression level of rat V1a receptor (V1aR) and V2 receptor (V2R) and eukaryotic 18S rRNA. The following TaqMan 18S rRNA control reagents and products for TaqMan gene expression assays were purchased from Applied Biosystems: rat V1aR, Rn00583910_m1; rat V2R, Rn00569508_g1; and 18S rRNA, 4319413E. To quantify the mRNA expression level for V1aR, V2R and 18s rRNA, relative standard curves were prepared using diluted cDNA from the outer medulla of rat kidney in triplicate (dynamic range, 1 to 1024-fold; 4-fold serial dilution). The slopes and R2 values of standard curve for V1aR, V2R and 18S rRNA were -3.346, -3.354 and -3.493, and 0.9984, 0.9993 and 0.9992, respectively. The amounts of V1aR and V2R were normalized to the amount of 18S rRNA.

### Statistics

Between two to five measurements were averaged to obtain a single value for each experimental condition in each tubule. The results were expressed as the mean ±SE. Student's *t* test or analysis of variance followed by the multiple comparison of Dunnet was employed for statistical analysis. Statistical significance was obtained at p<0.05. Dr. SPSS-II (Tokyo) was used for the analysis.

## Results

### Effects of AVP and ANP on PD and J T_CO2_ in lMALs and sMALs

**Figure 1:**
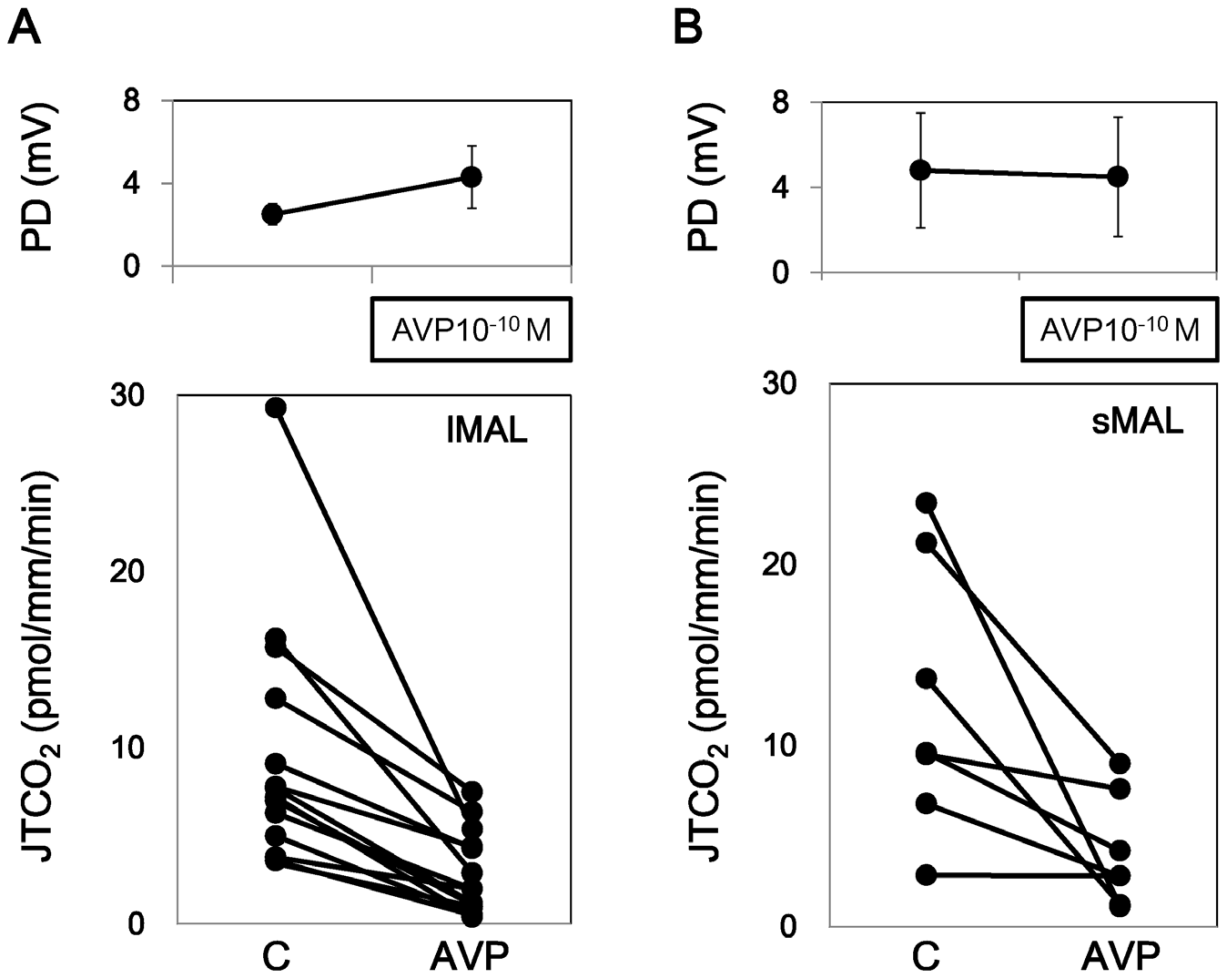
Effects of AVP on HCO_3_^−^ transport in lMAL and sMAL. The addition of 10^−10^ M AVP to the bath decreased HCO_3_^−^ absorption both in lMALs and sMALs, but the PD increased only in lMALs.

**Figure 2:**
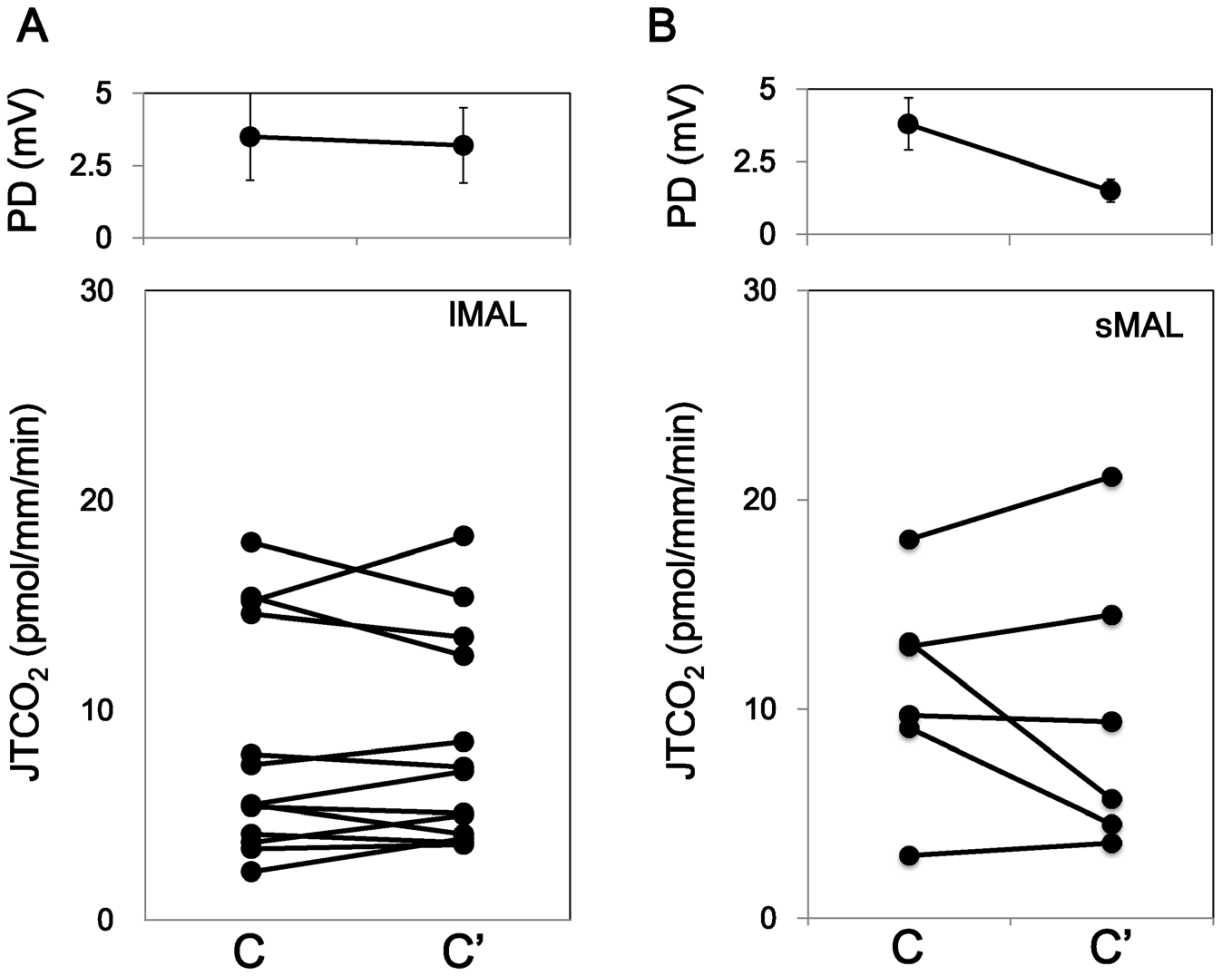
Time course of HCO_3_^−^ transport in lMAL and sMAL. Vehicle was added to the bath after the collection durng the control period. HCO_3_^−^ transport was stable with time both in lMALs and sMALs, although the PD was decreased only in sMALs.

We previously reported that AVP stimulated the PD in lMALs but not in sMAL. Our present data confirmed the effect of AVP on the PD. AVP increased the PD in lMALs, but it did not change the PD in sMAL. Time course of the basal condition showed no change in the PD in lMALs and a decrease in sMALs (Figs. 1 and 2 and Series 1 and 2 in Table 1). Time course experiments on the basal condition showed no change in HCO_3_^−^ reabsorption either in lMALs and sMALs (Fig. 2 and Series 2 in Table 1). AVP at a concentration of 10^−10^ M inhibited HCO_3_^−^ reabsorption both in lMALs and in sMALs (Fig. 1 and Series 1 in Table 1). These data are compatible with the report by Good et al. [13], [14].

**Table 1:** Effects of AVP, ANP and cGMP on HCO_3_^−^ transport in sMALs and lMALs.

	L (mm)	[TCO_2_]p (mM)		NCR (nl/mm/min)	[TCO_2_]c (mM)	J_TCO2_ (pmol/mm/min)	PD (mV)
Series 1 (C = control, E = 10^−10^M AVP)							
lMAL (n = 13)	0.63±0.10	23.7±1.0	C	2.68±0.47	19.4±1.0	9.6±1.9	2.5±0.5
			E	1.93±0.29	21.9±1.1	2.9±0.6*	4.3±1.5*
sMAL (n = 7)	0.71±0.08	26.3±0.6	C	2.85±0.64	21.3±0.7	12.4±2.6	4.8±2.7
			E	2.31±0.33	24.1±0.7	4.1±1.1*	4.5±2.8
Series 2 (C = control, E = control)							
lMAL (n = 13)	0.72±0.08	23.9±1.1	C	3.08±0.68	20.2±1.0	8.3±1.5	3.5±1.5
			E	2.13±0.24	19.8±1.0	8.3±1.4	3.2±1.3
sMAL (n = 6)	0.61±0.11	26.3±0.7	C	2.12±0.42	20.4±1.0	11.0±2.1	3.8±0.9
			E	1.99±0.38	21.1±0.4	9.8±2.5	1.5±0.4*
Series 3 (C, C′ = control, E = 10^−8^M AVP)							
lMAL (n = 7)	0.63±0.07	27.9±0.4	C	3.06±0.42	23.6±0.7	12.1±3.6	2.5±0.3
			E	2.56±0.27	24.8±1.0	8.3±3.0	1.8±0.3*
			C′	2.30±0.39	23.9±0.9	8.1±3.5	1.7±0.4*
Series 4 (C, C′ = 10^−10^M AVP, E = 10^−10^M AVP+10^−8^M ANP)							
lMAL (n = 8)	0.72±0.09	22.5±1.0	C	1.57±0.30	20.5±1.0	2.2±0.4	2.4±0.6
			E	1.90±0.45	19.3±0.9	4.7±0.7*	1.8±0.5*
			C′	1.40±0.14	20.0±1.1	2.6±0.6	1.6±0.5*
sMAL (n = 3)	0.69±0.08	27.5±0.1	C	2.77±0.39	26.4±0.3	2.5±0.6	2.7±0.2
			E	2.92±0.41	26.3±0.2	4.4±0.1*	1.9±0.2*
			C′	2.84±0.06	26.8±0.2	1.9±0.7	1.0±0.3*
Series 5 (C, C′ = 10^−10^M AVP, E = 10^−10^M AVP+10^−10^M ANP)							
lMAL (n = 8)	0.66±0.06	26.8±0.6	C	2.19±0.25	24.4±0.9	3.6±0.7	2.5±1.5
			E	3.21±0.41	24.4±0.7	6.5±0.8*	2.0±1.3
			C′	2.68±0.42	25.8±0.5	3.4±1.0	0.5±0.1*
Series 6 (C, C′ = 10^−10^M AVP, E = 10^−10^M AVP+10^−4^M cGMP)							
lMAL (n = 5)	0.58±0.09	26.6±0.8	C	2.22±0.58	24.0±0.4	4.0±0.3	1.7±0.8
			E	2.47±0.39	23.8±0.7	5.6±0.4*	1.1±0.7*
			C′	3.29±1.32	25.4±0.7	2.9±0.6	0.6±0.2

Values are mean ± SE. Abbreviations: L, tubular length; C, control period; E, experimental period; C′, recovery period; [T_CO2_]_p_, total CO_2_ concentration in perfusate and bath; [T_CO2_]_c_, total CO_2_ concentration in the collected solution; JTCO2, net bicarbonate absorption, NCR, normalized collection rate; PD, transepitherial potential difference;

* p<0.05 vs. control period.

**Figure 3:**
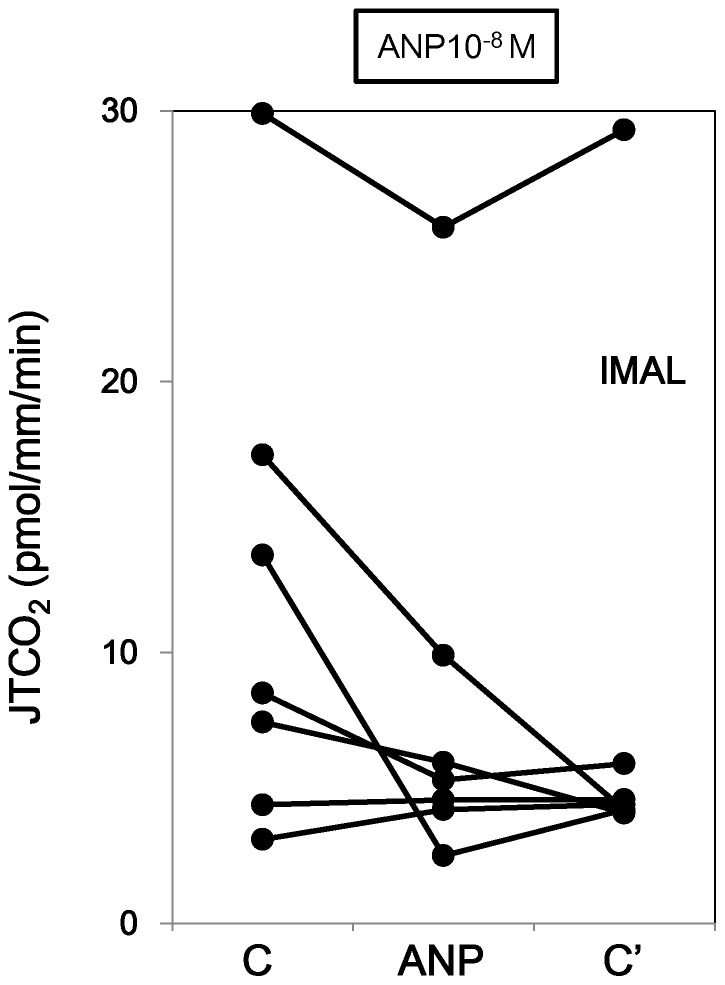
Effect of ANP on HCO_3_^−^ transport in lMALs in the absence of AVP. ANP at the concentration of 10^−8^ M did not cause changes in HCO_3_^−^ absorption in lMALs in the absence of AVP.

In contrast, ANP alone had no effect on HCO_3_^−^ transport in lMALs (Fig. 3 and Series 3 in Table 1).

### Effects of ANP and cGMP on the presence of AVP in lMALs and sMALs

**Figure 4:**
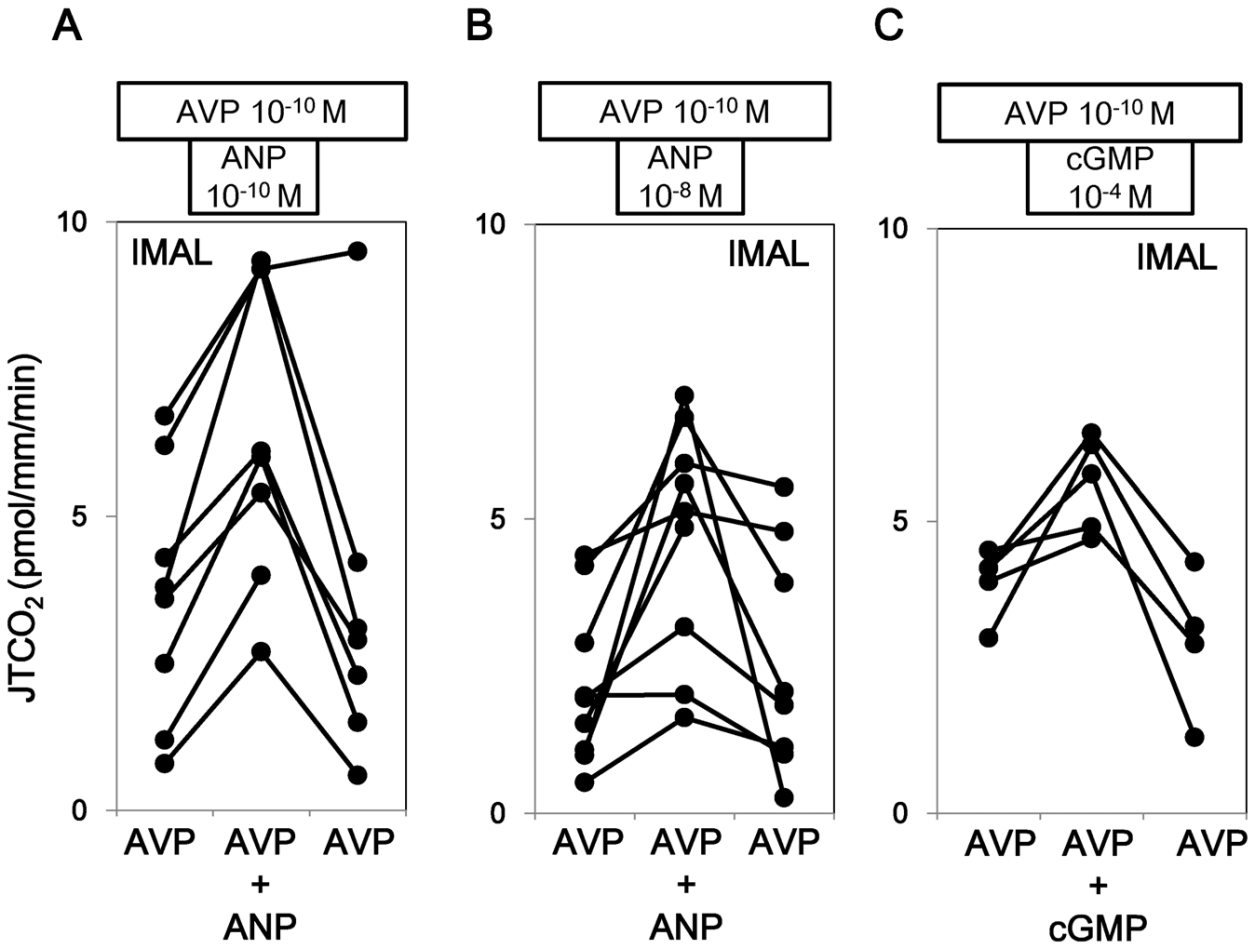
Effects of ANP and cGMP on HCO_3_^−^ transport in the presence of AVP in lMALs. ANP at the concentration of 10^−10^ M and 10^−8^ M significantly increased HCO_3_^−^ reabsorption in the presence of 10^−10^ M AVP in lMALs (A and B, respectively). cGMP at the concentration of 10^−4^ M mimicked the effect of ANP on HCO_3_^−^ transport in the presence of 10^−10^ M AVP in lMALs (C).

Next, the effect of ANP was examined in the presence of AVP. AVP at a concentration of 10^−10^ M was present in the bath throughout the experiment. ANP at a concentration of 10^−8^ M reversibly stimulated J T_CO2_ in lMAL (Fig. 4B and Series 5 in Table 1). The effects of low doses of ANP (10^−10^ M) and cGMP (10^−4^ M) were examined in lMALs. ANP at a concentration of 10^−10^ M also reversibly stimulated AVP-inhibited J T_CO2_ (Fig. 4A and Series 4 in Table 1). cGMP at a concentration of 10^−4^ M mimicked the effect of ANP on J T_CO2_ (Fig. 4C and Series 6 in Table 1).

**Figure 5:**
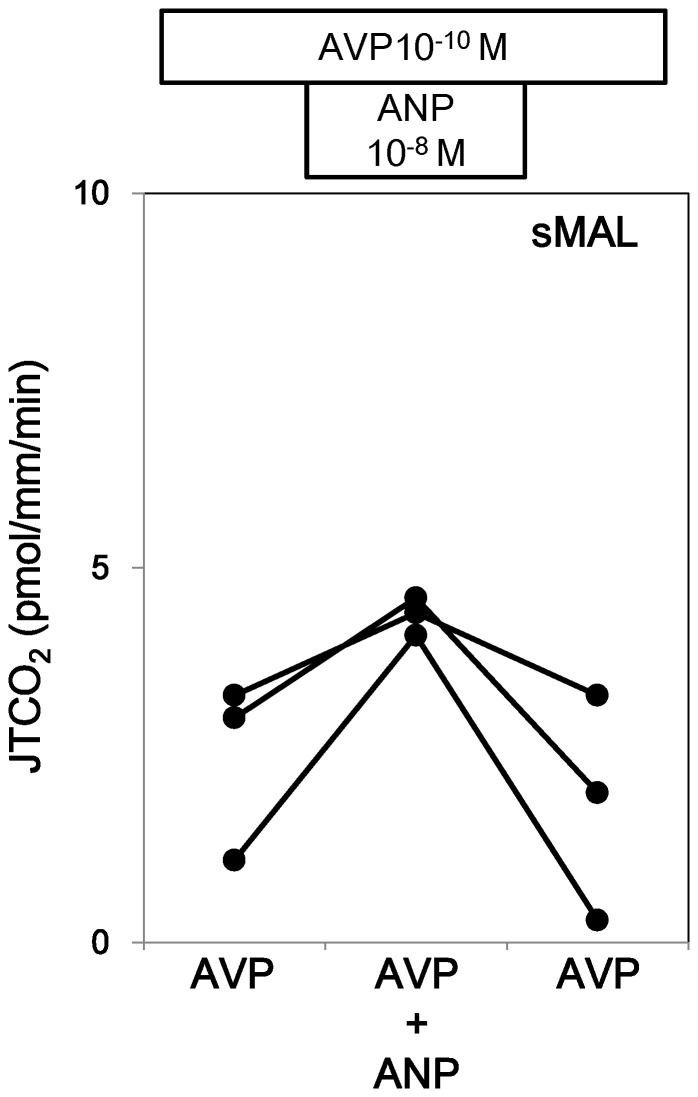
Effect of ANP on HCO_3_^−^ transport in the presence of AVP in sMALs. ANP at the concentration of 10^−8^ M stimulated HCO_3_^−^ reabsorption in the presence of 10^−10^ M AVP in sMALs.

ANP at a concentration of 10^−8^ M also reversibly stimulated J T_CO2_ in sMALs (Fig. 5 and Series 4 in Table 1).

### mRNA expression levels of vasopressin V1a and V2 receptors

**Figure 6:**
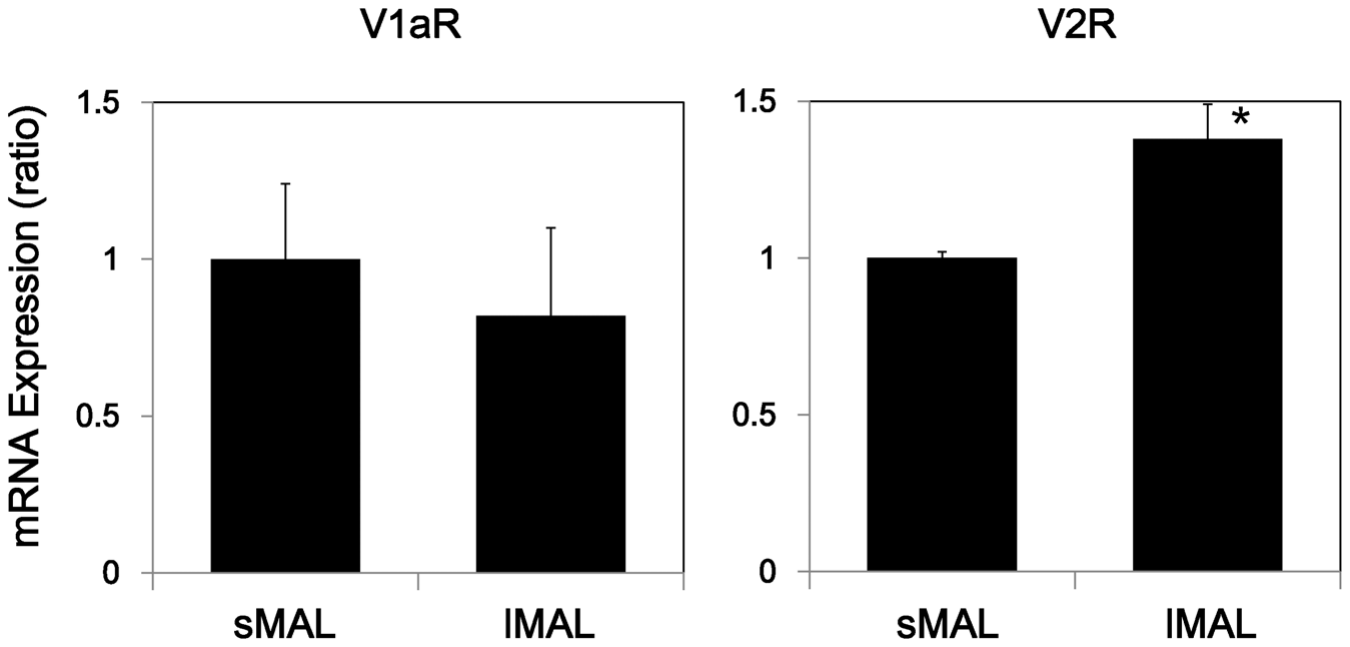
Expression levels of V1aR and V2R mRNA in sMALs and lMALs. The expression levels of V1aR in sMALs and lMALs were examined using real time PCR. sMALs and lMALs were dissected out of control rats after 30°C in a 0.1% collagenase solution in the presence of VRC. * p<0.05 vs. sMAL, n = 3–5.

The expression levels of V1aR and V2R in lMALs and sMALs were examined using real time PCR. V2R mRNA expression in lMALs was significantly higher than in sMALs (1.38±0.11* and 1.00±0.02 in lMALs and sMALs, respectively, n = 5, * p<0.05 vs. sMAL, Fig. 6). In contrast, V1aR mRNA expression was not significantly different between in lMALs and sMALs (0.82±0.28 and 1.00±0.24 in lMAL and sMAL, respectively, n = 3–4). The V2R/V1aR ratio in lMALs was higher than sMALs (1.7 and 1.0, in lMAL and sMAL, respectively, Fig. 6). To confirm the absence of possible contamination by outer medullary collecting ducts (OMCDs) in lMALs and sMALs, the V2R mRNA expression level was compared in OMCDs, lMALs, and sMALs. The expression of V2R mRNA was 10 times higher in OMCDs (9.60±0.74, n = 5) than in lMALs and sMALs, suggesting highly pure lMAL and sMAL samples.

## Discussion

Our data showed that AVP inhibited JT_CO2_ and that ANP counteracted the effect of AVP both in lMALs and sMALs. The effects of AVP and ANP opposed each other in lMALs and sMALs with respect to bicarbonate transport but only in lMALs with respect to chloride transport.

Acid-base regulation is an important role of the kidney alongside water and sodium excretion. Filtered bicarbonate from the glomerulus is completely reabsorbed by the nephron: 70–85% by proximal tubules, 10–20% by thick ascending limbs, 3–5% by distal convoluted tubules, and 2% by the collecting ducts [12]. HCO_3_^−^ reabsorption in the distal nephron is regulated by vasopressin and aldosterone [1], [12]–[18], [25], [26]. Because vasopressin and aldosterone are key factors to regulate body fluid, HCO_3_^−^ reabsorption in the MAL should be correlated with the maintenance of body fluid homeostasis. The effect of AVP on HCO_3_^−^ transport is different between the MAL and the collecting duct [12]–[18], [25]–[27]. AVP inhibits HCO_3_^−^ reabsorption in the MAL, while it stimulates reabsorption in the cortical collecting ducts. HCO_3_^−^ transport in the MAL has been extensively examined by Good and colleagues [13]–[18]. Vasopressin is known to inhibit HCO_3_^−^ transport in the MAL by regulating the Na^+^/H^+^ exchanger, NHE3, in the apical membrane [15], [17]. However, Good et al. reported that NHE1 in the basolateral membrane has a major role in basal HCO_3_^−^ transport in the MAL [15], [17].

AVP is known to induce NaCl reabsorption via NKCC2 in the MAL [5], [6]. We have shown that AVP stimulates Cl^−^ reabsorption in lMALs but not in sMALs, indicating the different effect of AVP on NKCC2 in lMALs and sMALs [11]. In contrast, HCO_3_^−^ transport in the MAL is not dependent on furosemide-sensitive NaCl reabsorption via NKCC2 [5], [27]. Inhibition of HCO_3_^−^ reabsorption by AVP and the counteracting effect of ANP on AVP occur both in lMALs and sMALs to the same degree. Thus, the regulation of HCO_3_^−^ transport and Cl^−^ transport by AVP and ANP are independent each other. Regulation of HCO_3_^−^ transport by AVP is caused by the activation of the Na-H exchanger (NHE) in the apical and basolateral membranes [15], [17], [18]. The lack of a difference in HCO_3_^−^ transport between lMALs and sMALs suggests that the NHE in lMALs and sMALs are functionally same, whereas the NKCC2s in lMALs and sMALs are functionally different. It would be interesting to compare the distributions of NKCC2 isoform A, B, and F between lMALs and sMALs.

The diuretic action of ANP is now used clinically. ANP has diuretic and natriuretic effects in various segments of the nephron including the MAL [1], [11], [19]–[21], [28]–[32]. The effects of ANP on sodium excretion are mainly caused by the action in the thick ascending limb and collecting duct [1], [11], [20], [21], [29]–[32]. The effects of ANP on glomerular filtration and proximal tubule sodium transport have been less well studied [1], [33]. Our data show that ANP participates in the regulation of not only NaCl excretion but also acid-base balance in the MAL. In our experiments, ANP did not have a direct effect on HCO_3_^−^ transport in the MAL. Stimulation of HCO_3_^−^ transport by ANP was observed only in the presence of AVP, suggesting that ANP might inhibit the vasopressin-dependent protein kinase A (PKA) pathway. Vasopressin stimulates cAMP generation and subsequent PKA activity via V2R by activating adenylyl cyclase, while ANP stimulates cGMP generation [19], [20], [34]. We showed that cGMP mimicked the effects of ANP in the MAL in the presence of AVP. Good et al. have shown that PGE2 stimulates HCO_3_^−^ reabsorption in the presence but not in the absence of AVP [16]. The effects of PGE2 and ANP look the same in terms of their inhibitory effects on the vasopressin-induced decrease in bicarbonate transport. However, the effects of ANP and PGE2 on AVP-dependent cAMP generation are different [11]. We have shown that ANP does not change AVP-dependent cAMP accumulation [21], whereas PGE2 inhibits vasopressin-dependent cAMP generation in the MAL [11]. It is hypothesized that PGE2 inhibits upstream of PKA whereas ANP inhibits downstream of PKA pathway. AVP is known to stimulate the PKC pathway via V1aR in the intercalated cells of the collecting ducts [35]. Activation of PKC by luminal AVP causes a decrease in the AVP-dependent cAMP accumulation in initial inner medullary collecting duct [34]. If ANP stimulates PKC activity, a decrease in AVP-dependent cAMP accumulation would occur. Therefore, the oppositional effect of ANP on AVP action would be PKC independent in the MAL.

Our data showed that lMALs express V2R more than do sMALs. V2R has a major role in the antidiuretic action of AVP and is present in lMALs, sMALs and the principal cells of the collecting ducts [1], [34], [36]. In contrast, V1aR is present in lMALs, sMALs, and the intercalated cells of the collecting duct and has an important role in V2R-mediated vasopressin effects, including on HCO_3_^−^ transport [1], [23], [37]–[40]. How V1aR and V2R participate in HCO_3_^−^ transport in the MAL is not clear yet [1], [23], [38]–[40]. In the collecting duct, vasopressin stimulates acid excretion in the intercalated cells by regulating the action of aldosterone [23]. V1aR is essential for the nucleocytoplasmic transport of mineralocorticoid receptor in the intercalated cells [35]. The lack of V1aR causes type 4 renal tubular acidosis [23]. Although mineralocorticoid receptorsare present in the MAL, the nature of interaction between V1aR and mineralocorticoid receptors in the MAL is not yet known [41], [42]. Good et al. have reported nongenomic regulation of NHE3 by aldosterone in the MAL [18]. The present study may imply a different mechanism of HCO_3_^−^ transport by AVP in the MAL compared with the collecting duct because both V1aR and V2R are present in a single type of cell in the MAL. The role of V1aR in HCO_3_^−^ transport in the MAL and its interaction with aldosterone and ANP needs to be examined further.

In conclusion, the present study clearly shows that AVP inhibits HCO_3_^−^ reabsorption in both lMALs and sMALs and that ANP counteracts the action of AVPon HCO_3_^−^ reabsorption in both lMALs and sMALs.
